# OA11.04. Psychosocial outcomes from the chiropractic and integrative care low back pain randomized clinical trial

**DOI:** 10.1186/1472-6882-12-S1-O44

**Published:** 2012-06-12

**Authors:** K Westrom, G Bronfort, R Evans, M Maiers

**Affiliations:** 1 Northwestern Health Sciences University, Bloomington, USA

## Purpose

Evidence to date suggests there is no one treatment approach which is clearly superior for low back pain (LBP). It is plausible that combining treatments with small to moderate effect sizes will improve patient outcomes. Additional benefit might also be achieved by individualizing treatment plans according to patient presentation, for example, by addressing psychosocial factors. Our objective was to assess the relative clinical effectiveness of: 1) mono-disciplinary chiropractic care versus 2) multi-disciplinary integrative care for LBP, within the context of defined clinical care pathways.

## Methods

Adult patients with LBP rated 3 or greater (0-10) for at least 6 weeks were randomized to one of two care teams for 12 weeks of treatment. Integrative multi-disciplinary care included acupuncture and Oriental medicine, cognitive behavioral therapy, exercise, massage, chiropractic and/or medicine. Mono-disciplinary care consisted of chiropractic alone. After randomization, psychosocial measures were used to guide treatment suggestions. The primary outcome was self-rated pain. The secondary outcomes discussed here are: fear avoidance, quality of life, self-efficacy, kinesiophobia and passive and active coping.

## Results

A total of 201 participants were randomized. There were no significant between-group differences in kinesiophobia, fear avoidance beliefs and self efficacy at any time point. Patients randomized to mono-disciplinary chiropractic care were more likely to report passive coping at week 12 compared to the integrative group. At week 52, patients in integrative care rated quality of life significantly better than the mono-disciplinary group.

## Conclusion

Multi-disciplinary integrative care did not yield significant changes in self-rated psychosocial measures compared to mono-disciplinary chiropractic care alone. Although at one year patients from the integrative group rated quality of life significantly higher, it is important to consider this in the context of utilization. Upcoming cost effectiveness data analysis will aid in the interpretation of these results.

